# Happy to help—if it’s not too sad: The effect of mood on helping identifiable and unidentifiable victims

**DOI:** 10.1371/journal.pone.0252278

**Published:** 2021-06-01

**Authors:** Hagit Sabato, Tehila Kogut

**Affiliations:** 1 School of Education, The Hebrew University of Jerusalem, Jerusalem, Israel; 2 Department of Education, Ben Gurion University of the Negev, Beersheba, Israel; Middlesex University, UNITED KINGDOM

## Abstract

People’s preference to help single victims about whom they have some information is known as the *identifiable victim effect*. Previous research suggests that this effect stems from an intensive emotional reaction toward specific victims. The findings of two studies consistently show that the identifiability effect is attenuated when the subject is in a positive mood. Study 1 (along with a pilot study) demonstrate causal relationships between mood and identifiability, while using different manipulations to induce moods. In both studies, donations to identified victims exceeded donations to unidentified people—in the Negative Mood manipulations—while participants in the Positive Mood conditions showed no such preference. In Study 2, individual differences in people’s moods interacted with the recipient’s identifiability in predicting donations, demonstrating that the identifiability effect is attenuated by a positive mood. In addition, emotional reactions toward the victims replicate the donation pattern, suggesting emotions as a possible explanation for the observed donation pattern.

## Introduction

Imagine that your country’s national team is competing against another country’s national team in the World Cup finals. After a nerve-racking and exciting game, your country wins! People dance in the streets, and everyone is in a great mood. How do you expect these happy people to react to a beggar who is standing by the roadside, begging for money? More generally, is this the right time for charity organizations to ask for donations?

Research on the *identifiable victim effect* demonstrates that people are more generous toward single, identifiable victims (about whom they have some information) than toward unidentifiable, abstract ones [1–4]. This line of research suggests that feelings evoked by considering the victim’s plight play a major role in explaining this effect [3]. Specifically, Kogut and Ritov [1] have shown that when asked about their distress at learning of the plight of certain victim(s), participants who had been told about a single identified victim rated their distress higher than those who had been told about an unidentified victim or a group of victims (whether identified or not). Indeed, evidence from neural research suggests that emotions play a major role in decisions regarding donations to a single identified victim, while they have a smaller effect on donations to charitable organizations [5].

However, interest in, and openness to, the distress triggered by a single identified victim may be dependent on the prospective donor’s mood when encountering the donation request. Specifically, building upon *mood maintenance theory* [6–8], and *the Negative* state *relief model* [9–11] we hypothesized that people in a negative mood are more likely to sympathize with a specific identified victim, and may see the identifiable case as a tangible opportunity to help and improve their own mood. Conversely, people who are in a positive mood may prefer to avoid hearing the story of a specific identified victim, fearing that this might ruin their positive mood as we further discuss. In the present research we therefore set out to examine our hypothesis that the identifiable victim effect is attenuated under a positive mood. Research in the past few decades on the effect of moods on generosity has yielded mixed results. On the one hand, people tend to be more generous and friendly and tend to help more when they are in a positive mood (compared with controls) [12–15]. This effect has been replicated in relation to various helping behaviors—e.g. contributing or lending money [14, 16]; donating blood [17]; and helping the experimenter [18, 19]—as well as under various mood-enhancing methods, such as listening to a comedy [14]; recalling and writing about happy experiences [17, 20]; and experiencing success [12, 19].

On the other hand, there is evidence that, in certain circumstances, a negative mood is also likely to enhance helping [9, 20–22] These conflicting findings are discussed in the literature in relation to various cognitive mechanisms that may also play a role in the context of prosocial behavior—such as affect as information [23–26]; attention and information processing styles [27–29]; and the effect of mood on people’s sense of efficacy [30]. The *dual-process approach* to decision-making may offer another explanation for the effect of emotions on prosociality. This model links emotions to intuitive, automatic processes (as opposed to more controlled, deliberative ones), whereby people are more likely to use heuristics in their behavior [31–33]. Although studies of the effect of emotions on altruistic behavior have yielded somewhat inconsistent results [34, 35], the tendency to use heuristics has been found to be related to prosocial behavior in various contexts (e.g. cooperation—[36]; altruistic punishment—[37]; rejecting instrumental harm—[38]; and speciesism—[39]; see [40] for a review). Similarly, intuitive, automatic reactions have been linked to several systematic biases in helping decisions, such as the *Proportion dominance* effect [41]; the Recency effect [42]; the *charity beauty premium* effect [43]; and *scope neglect* [44]. The identifiable victim effect is another example of the effect of an intuitive, automatic response, that may result in decisions that deviate from a rational, deliberate reaction [4].

Since the identifiable victim effect has been mostly explained by the spontaneous emotional arousal toward the victim [1, 2, 4, 45], emotional mechanisms—specifically mood maintenance theories—appear to be particularly relevant to explaining the possible effect of mood on the identified victim effect. According to mood maintenance theories (which emphasize the anticipated emotional consequences of behaviors) people in a positive mood tend to seek out positive activities that maintain or enhance their good mood—and therefore will only help others when this is not too costly to them, or not likely to ruin their good mood [7, 11, 46–49]. By the same token, people mired in a negative mood may help others in a bid to improve their own mood (e.g. “the negative state relief model” [9–11]. However, since people tend to be less responsive to others overall while they are in a negative mood, they are most likely to help when the helping situation is salient—such as a highlighted or a vivid request [50].

Pursuant to this line of research, Andrade and Cohen [46] have suggested that informational (affective evaluation) and goal-directed (affective regulation) explanations for mood effects on behavior may be reconciled by understanding the dominant mechanisms behind a helping situation. For example, when a behavioral activity has no clear mood-changing property, people in a negative mood are less likely to be motivated to act, since no mood enhancement is anticipated. However, when the mood-changing properties of a behavioral activity is salient—such as in the case of a donation to an identified victim (who provides a concrete opportunity to help, and feel meaningful) affect regulation may be a dominant impulse—enhancing prosocial behavior among participants in a negative mood more than those in a positive mood. However, for participants in a positive mood (who are not looking for opportunities for mood improvement), being presented with an identified victim may trigger the anticipation of a negative mood—thereby probably activating self-protective regulatory mechanisms, including the avoidance of requests to help. This is less likely to occur when the help target is more general and abstract, however, as it offers the prospective donor the warm-glow feeling of helping, without the cost of negative emotions. The above literature suggests, therefore, that helping behavior may be best predicted by the interaction between the perceiver’s mood and the characteristics of the helping situation—such as the difficulty of helping, the salience of the need, and most importantly the extent to which helping is likely to affect the perceiver’s mood later. Helping behavior may also be predicted by the interaction between the perceiver’s mood and the attributes of the intended help recipients—such as the extent to which they induce positive versus negative emotions.

### The present research

Based on the literature that suggests that the increased incidence of helping induced by a positive mood may be limited to situations that are not expected to spoil the helper’s mood [47], we hypothesized that although people in a positive mood tend to behave in an overall more prosocial way, they would be less responsive to a single identified victim and may indeed emotionally detach themselves from such situations—because sad stimuli of this sort tends to evoke distress and is likely to ruin their mood. Moreover, when they are in a positive mood, people are more likely to comply with a general request for help that requires less emotional involvement—as this provides an opportunity to feel meaningful without an emotional cost.

Conversely, since people in a negative mood tend to be more responsive to the needs of others especially when the helping situation is salient [50], the single identified victim is more likely to attract their attention. People in a negative mood are more able to relate to and sympathize with an identified victim, as such a victim provides a specific, clear target for their emotions and may offer a tangible opportunity to help and to improve their mood if they do. Lastly, since the *Identifiable Victim Effect* has been found in previous research to pertain irrespective of the prospective donors’ mood [1, 2], we expect people who are in a comparatively neutral mood to exhibit it (much like those who are in a negative mood). This is because, in the absence of the motivation to avoid the sad stimulus (that mostly exists among people who are in a positive mood), a single identified victim tends to elicit a spontaneous response of closeness and empathy [3, 44].

Taking all these considerations into account, we suggest that mood may moderate the identifiability effect, such that the effect is attenuated under a positive mood. This hypothesis is in line with research that examined the role of a chronic (as opposed to an incidental) emotional state in mediating the identifiable victim effect [51]. In that study, people with a secure attachment style showed no preference for helping a specific identified victim over a group of victims, while the identifiability effect was strongest among people with an anxious attachment style who felt greater similarity and connectedness to identified single victims.

Moreover, in line with the *mood maintenance theory*, we suggest that the attenuation of the identifiable victim effect under a positive mood may stem from happy people’s reluctant to spoil their mood by contemplating the sad case of an identified victim. Therefore, the emotional reaction toward identified victims is expected to be weaker under a positive mood (compared with a sad or neutral one), which in turn would curb the tendency to increase donations to identified victims.

We examined these hypotheses in two studies (followed by an exploratory pilot study). In the first study, we manipulated participants’ moods experimentally, and examined their donations to identified victims and to generic victims with the same need—thereby demonstrating a causal relationship between mood and the *identifiable victim effect*. In Study 2, we sought to examine our hypothesis in a more naturalistic setting, using individual differences in participants’ ongoing moods, after which they read about a single identified ill child or about sick children in general, and asked about their willingness to donate and about their emotions toward the victim(s).

The three experiments were approved by Ben-Gurion University, Department of Education Ethics committee. All participants were adults and have signed a written consent before their participation in the studies.

### Pilot study

We began our investigation with an explorative pilot study, in which 95 undergraduate students (84% women, mean age = 23.45, SD = 1.20) were randomly assigned to one of four experimental conditions of a 2x2 design, that manipulated the identifiability of the target (*Single Identified* versus *General Need*) and mood (*Positive* versus *Negative*). Participants were then asked to write about a recent event that had made them happy or sad (manipulated between-subjects) in a personal manner, as though telling it to a good friend [e.g. 52].

Next, all participants completed a PANAS questionnaire, in which they rated the extent to which they felt about each of 20 feelings and emotions (10 positive and 10 negative ones). Next, they were randomly assigned to one of two experimental conditions, which manipulated the intended beneficiary of a donation request: a single identified sick child (hereafter, *Identified*) or sick children in general (hereafter, *General Need*). All participants were presented with the same basic story, adapted from Kogut and Ritov [1], that described sick children in need of an expensive medicine. Participants in the Identified condition were told that the child in question was Dan—a three-year-old boy undergoing treatment at a medical center, whose life is in danger—along with a photograph of him. In all our studies, the child in the photo appeared with a neutral expression, to avoid possible interactions between the participant’s mood and that of the child. Participants in the General Need condition were told the victims in question were a group of sick children being treated at a medical center whose lives are in danger—with no further identifying information. Next, all participants read that a new drug that may cure the disease has recently been developed—however, it is extremely expensive, and not yet covered by medical insurance. Finally, participants were asked to imagine that they had a sum of NIS 100 (~$31) in their wallet, and to indicate how much they were willing to donate from it to the child or children—ranging from *0* (“I would not like to donate”) to the full amount.

### Results

We first examined the extent to which mood manipulation affected the ratings of positive and negative emotions (PANAS) and mood. Results of a mixed-model ANOVA with Positive and Negative emotions as a within-subject variable and Mood manipulation as a between-subject variable revealed the expected significant interaction, F(1, 93) = 14.72, p < .001, such that positive emotions were rated significantly higher in the Positive Mood condition (M = 2.98, SD = 1.00) than in the Negative Mood one (M = 2.40, SD = .72; F(1, 93) = 10.52, p = .002), while the opposite pattern occurred for negative emotions which were significantly higher in the Negative Mood condition (M = 2.57, SD = .82) than in the Positive Mood condition (M = 2.00, SD = .87; F(1, 93) = 10.42, p = .002). Finally, mood ratings were also significantly higher in the Positive Mood condition (M = 7.54, SD = 1.63) than in the Negative Mood one (M = 4.76, SD = 1.58; t(93) = -8.42, p < .001, d = 1.65).

The participants’ willingness to donate (WTD) ranged from *0* (5 participants) to *100* (16 participants); Mean = 45.23, SD = 31.03. Results of a two-way ANOVA of WTD as a function of mood and identifiability revealed no significant main effects. However, the interaction between mood and identifiability was significant: F (1, 91) = 4.03, p = .048, *η*_*p*_^*2*^ *=* .042. Simple effect analysis reveals that the participants’ willingness to donate to a single identified victim (M = 51.25, SD = 26.09) exceeded that in relation to the general need (M = 34.76, SD = 27.95) in the Negative Mood condition (F (1, 91) = 3.21, p = .067, *η*_*p*_^*2*^ *=* .03). However, in the Positive Mood condition, donations to the general need (M = 51.46, SD = 32.88) and to the single identified victim (M = 42.50, SD = 34.68) did not differ significantly, and tended to be higher in the General Need condition (F (1, 91) = 1.06, p = .31, *η*_*p*_^*2*^ *=* .011).

The results of the pilot study provide initial evidence in support of our prediction that the identified victim effect is attenuated under a positive mood. While participants in the induced negative mood condition exhibited the identified victim effect and were more willing to donate to identified victims than to general ones, participants in the induced positive mood induction condition showed no such preference. We therefore designed Study 1 to further examine our hypothesis while adding a control condition with no mood-enhancement manipulation, and soliciting actual donations (rather than merely asking the participants about their willingness to donate).

### Study 1

#### Method

One hundred and ninety-seven undergraduate students (61% women, mean age 23.53, SD = 2.41) took part in the study in return for NIS 15 (~ US $4) in the form of one NIS 5 and one NIS 10 coin. Sample size was predetermined based on the results of the pilot study. In study 1, the partial eta square of the interaction in question was *η*_*p*_^*2*^ *=* .042 which translates to f = .21. Using G*Power3 [53], with small to medium effect size (f (2) = .21), power = 0.80, α = .05. Participants were randomly assigned to one of 6 experimental conditions of a 2X3 design, in which the identifiability of the target (*identified* versus *general need*) and mood (*negative*, *positive* and *control*) were manipulated. Participants were then asked to evaluate two short videos, supposedly in preparation for a future study. After entering the lab, they were each seated in an individual cubicle before a private computer screen, where they watched two video clips (each lasting about 3.5 minutes) aimed at inducing the same type of emotion (two negative, two positive, or two neutral clips). The clips were adopted from Harle and Sanfey [54], who chose them from a pool of several other clips, following a pilot study that had gauged how successful they were in inducing the above moods. After watching the two videos, participants answered a few questions about them—such as *Which of the two was more interesting*?, *How enjoyable was each clip*?, etc. (to increase reliability). Next, participants completed the PANAS questionnaire and rated their mood (as in the pilot study). Finally, they were told that the lab was collaborating with the *Hayim* charitable organization in aid of sick children, whose message they were asked to read. The message was about either a single identified child or a group of unidentified children in need of an expensive medication (manipulated between subjects, as in the pilot study). After reading about the children’s plight, participants were asked whether they were willing to donate money to help save their lives. If they agreed, they could contribute any amount they wished. Specifically, they could donate any or all of the NIS 15 fee they had received for their participating in this study, or a greater amount, by adding to it as much as they chose. They were then instructed to place the completed questionnaire, with their donation (if any), in a sealed, unmarked envelope. (All the money raised in this study was subsequently donated by the experimenter to an Israeli organization that helps children with cancer).

### Results

We first examined the extent to which the mood manipulation affected ratings of PANAS and mood. Results of a mixed model ANOVA with Positive and Negative emotions as a within subject variable and Mood manipulation as a between subject variable revealed the expected two way interaction F(2, 188) = 48.27, p < .001. Specifically, a one-way ANOVA of positive emotions (mean ratings of the positive items) as a function of mood, revealed a significant difference between the three groups F (2, 188) = 20.46, p < .001 *η*_*p*_^*2*^ *=* .18. Post-hoc comparisons revealed that positive emotions were rated significantly higher in the Positive Mood condition (M = 3.17) than in the two other conditions (negative, M = 2.46, p < .001; and control M = 2.47, p < .001)—while the two latter conditions did not significantly differ (p = .98). Similarly, the difference in the ratings of negative emotions was also significant between the three groups F(2,188) = 32.86, p < .001, *η*_*p*_^*2*^ *=* .26. Post-hoc comparisons reveal that negative emotions were rated significantly higher in the Negative Mood condition (M = 2.58) than in the two other conditions (positive, M = 1.57, p < .001; and control M = 1.75, p < .001), while the two latter conditions did not significantly differ (p = .19). Finally, mood ratings were also significantly different in the three conditions, F(2, 188) = 13.22, p < .001, *η*_*p*_^*2*^ *=* .12. Post-hoc comparisons reveal that mood ratings were rated significantly better in the Positive Mood condition (M = 7.94) than in the two other conditions (negative, M = 6.43, p < .001; and control, M = 7.13, p = .008), the two latter conditions also significantly differ (p = .02). We therefore concluded that the movies were successful in inducing positive, negative and neutral moods.

**Fig 1:**
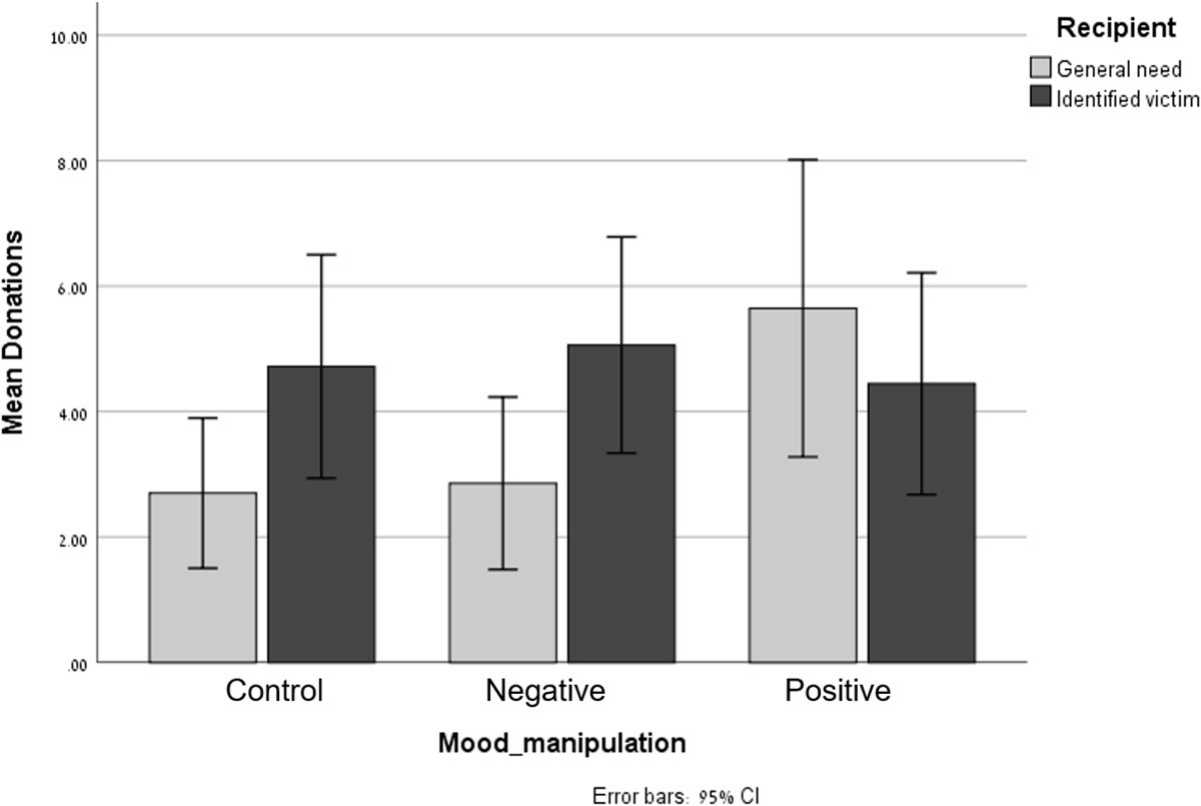
Donation amounts to identified targets and to general ones as a function of mood.

Participants’ donations ranged from nil (68 participants) to NIS 20 (3 participants); Mean = 4.23, SD = 4.94. Results of a two-way ANOVA of donation amounts as a function of Mood and Identifiability revealed no significant main effects. The interaction between Mood and Identifiability approached significance F (2, 191) = 2.54, p = .08, *η*_*p*_^*2*^ *=* .026. As can be seen in Fig 1, participants’ donations to the single identified victim (M = 4.72; SD = 4.94) exceeded donations to the general need (M = 2.70, SD = 3.20) in the control condition (this difference approached significance, F (2, 191) = 2.64, p = .10, *η*_*p*_^*2*^ *=* .014) and in the Negative Mood condition (M = 5.06, SD = 4.86 in the Identified condition, and M = 2.86, SD = 4.00 in the General Need condition; F (2, 191) = 3.45, p = .06, *η*_*p*_^*2*^ *=* .018); while in the Positive Mood condition, donations to the general need (M = 5.64, SD = 6.45) and to the single victim (M = 4.44, SD = 5.22) did not significantly differ, and tended to be higher in the General Need condition (F (2, 191) = 1.01, p = .32, *η*_*p*_^*2*^ *=* .005).

Since our hypothesis was that the Identifiable Victim Effect would occur in the Negative mood and Control conditions—but not in the Positive mood condition—we conducted another ANOVA on the amount of money donated, with two levels of moods: the Negative and the Control conditions (in which we predicted to find the Identifiable Victim Effect) versus the Positive mood condition (in which no identifiability effect was expected). The other independent variable was the victim’s identifiability. Results reveal a significant interaction between Mood and Identifiability, F (1, 193) = 5.10, p = .025, *η*_*p*_^*2*^ *=* .026. Specifically, simple effect analysis revealed a significant difference between donations to the identified victim and to the generic ones under the Negative+Control condition F (1, 193) = 6.11, p = .014, *η*_*p*_^*2*^ *=* .03, but not in the Positive mood condition F (1, 193) = 1.01, p = .32, *η*_*p*_^*2*^ *=* .005.

The results of Study 1 replicated the pattern found in the pilot study, while soliciting real donations (as opposed to merely asking the participants if they were willing to do so). The identifiable victim effect (i.e. the tendency to donate more to an identified victim than to a general cause of helping victims in the same predicament), was evident in the Negative Mood and in the control conditions, but was attenuated in the Positive Mood condition—with donations to the General Need tending to be somewhat higher than to the single identified victim.

One limitation of Study 1 is that variance in participants’ initial mood may have weakened the effect of the priming manipulation. Therefore, in Study 2, we aimed to further examine the hypothesis that the identifiable victim effect attenuates under a positive mood, while measuring individual differences in participants’ natural, ongoing moods, rather than experimentally manipulating them. Examining the relation between participants’ actual mood and their donations to the two types of victims also has the potential to increase the ecological validity of the study. In addition, in this study we asked participants about their emotional reaction toward the victim(s), to examine the hypothesis that identifiability increases the emotional arousal toward the victim when the subject is in a negative or a neutral mood, but less so if they are in a relatively positive mood.

### Study 2

#### Method

Two hundred and eight undergraduate students—77% women, mean age = 24.73 (SD = 1.49)—took part in the study, in return for an entry into a raffle in which one in twenty participants won NIS 100 (~ $25). Sample size was predetermined as in Study 1.

Participants were given a short questionnaire, which they were asked to complete in the order of its questions. On page 1, they were asked to state their current mood, on a 10-degree scale ranging from *1* (“Very bad”) to *10* (“Very good”). Next, they completed a PANAS questionnaire (as in the previous studies). On the following page, they were randomly assigned to one of two experimental conditions, which manipulated the intended beneficiary of a donation request: a single identified sick child, or sick children in general (using the same stimuli as in the previous studies). After reading about the children’s plight, participants were told that the lab was working with a charity organization which is raising money to help save the victims’ lives, and asked whether they would be willing to donate any part of their NIS 100 prize, if they won it in the study raffle—and if so, how much (it was made clear that winners would be asked to make good on the pledge). Lastly, participants were asked to rate their feelings of distress from, and concern for, the sick children on a 7-point scale, using two items (adapted from [1]). The first sentence, which gauged feelings of distress, read: “*After reading the child’s [children’s] story I felt worried*, *upset and sad*.*”* The second sentence, examining feelings of empathic concern, was: “*I felt sympathy and compassion towards the sick child [children]*.” The 7-point response scale for each sentence ranged from “Not at all” to “Very much.”

## Results and discussion

**Fig 2:**
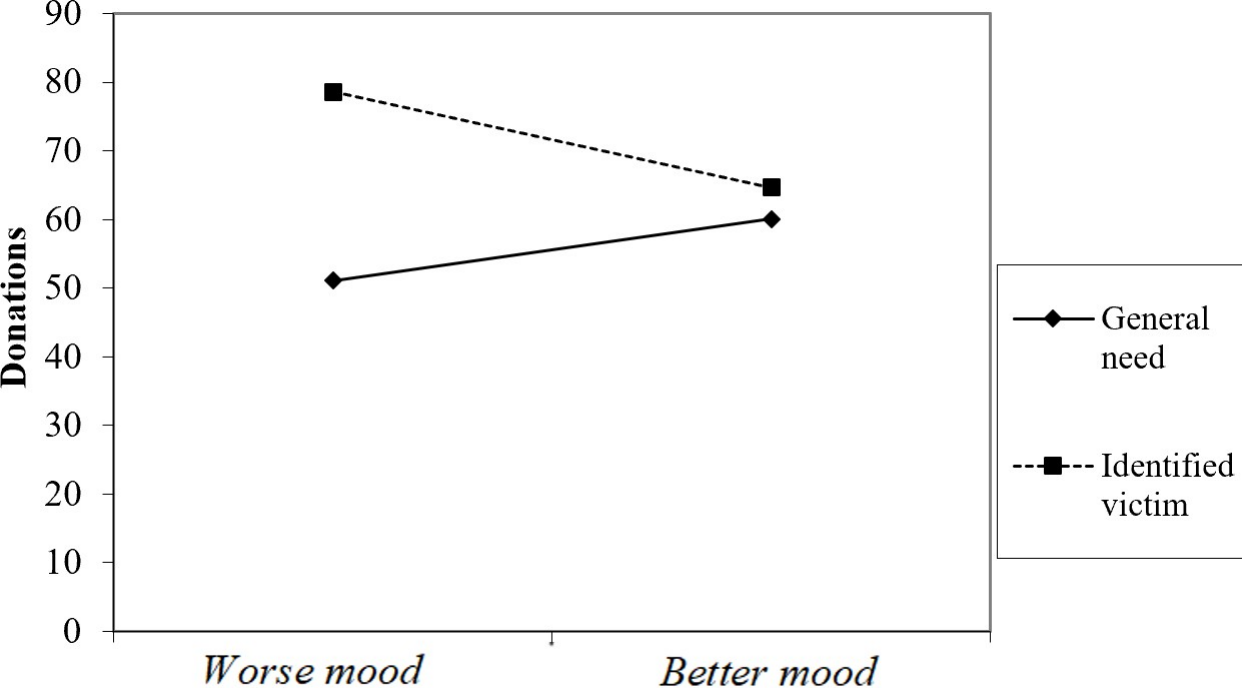
Donation amounts to identified victims and to general ones, as a function of reported ongoing moods.

The participants’ donations ranged from nil (20 participants) to NIS 100, *M* = 63.65, *SD* = 37.25. To examine the role of mood, Identifiability, and the interaction between them in predicting donations, a simple regression analysis was conducted on donation amounts. The model was significant F(3, 202) = 5.18, p = .002, r^2^ = .071. Specifically, the role of the target’s identifiability was significant (*t* = 2.96, *β* = .83, *p* = .003)—such that overall, identified recipients received higher donations (M = 71.59, SD = 33.31) than unidentified recipients in the General-Need condition (M = 55.47, SD = 39.42). In addition, the interaction between the participants’ mood ratings and Identifiability was significant (*t* = -2.26, *β* = -.66, *p* = .025). This interaction was plotted in Fig 2 as recommended by Aiken and West [55]: one SD above the mean of the mood scale, and one SD below that mean in each condition (Identified and General Need). As illustrated in Fig 2, in the Identified condition, worse moods were significantly linked to higher donations (*t* = -2.19, *β* = -.21, *p* = .031). Conversely, in the General Need condition, no significant link was found between mood and donations (and higher donations tended to correlate with better moods—*t* = 1.13, *β* = .11, *p* = .26).

Ratings of positive and negative emotions significantly correlated with mood (r = .52, p = .001 and r = -.48, p = .001 respectively). A simple regression analysis on donation amounts—with the difference between positive and negative moods, Identifiability, and the interactions between them—revealed similar results to the regression with mood ratings reported above. Specifically, the interaction between Identifiability and the difference between positive and negative moods was significant (*t* = -2.14, *β* = -.22, *p* = .033).

We next turned to the participants’ emotions toward the victim(s). The two items (Distress and Concern) were highly correlated (α = .81), and were averaged to form a single emotional reaction scale. A regression analysis on this scale with Mood ratings, Identifiability, and the interaction between them on the Emotions scale, revealed significant results, F (3, 203) = 3.21, p = .024, r^2^ = .05. Specifically, the role of recipient’s identifiability was significant (*t* = 2.91, *β* = .83, *p* = .004). In addition, the interaction between Mood and Identifiability was also significant, replicating the pattern found for donation amounts—such that, in the Identified condition, negative moods were significantly linked to stronger emotions (*t* = -2.73, *β* = -.26, *p* = .007), while in the General Need condition, no significant link was found between mood and emotions (*t* = 1.45, *β* = .14, *p* = .15).

Based on these results, we predicted that Emotions toward the victim(s) mediate the link between Identifiability and donations—but only under negative moods, but not positive ones. To test this hypothesis, we used the SPSS PROCESS macro model 7, with bootstrap techniques and 5,000 resamples [56]. We predicted that the conditional indirect effect of Identifiability on Donations through Emotions (as a mediator) occurs only under negative moods (and not under positive ones; moderator). As previously noted, the interaction between Identifiability and Mood significantly predicted Emotions (t = -3.00, p = .003, 95% CI [-.64, -.13])—which in turn significantly predicted Donations (t = -5.78, p < .001, 95% CI [5.85, 11.90]). Emotions mediated the effect of Identifiability on Donations overall (B = -3.45, SE = 1.40, 95% CI [-6.29, -.83). Most importantly, the mediating role of Emotions was significant only in lower levels of the mood scale (i.e. negative mood; B = 5.83, SE = 3.02, 95% CI [.26, 11.88), but not at the higher levels of the scale (i.e. more positive mood; B = -4.52, SE = 2.69, 95% CI [-10.03, .51)—suggesting that Emotions mediate the interaction between Mood and Identifiability on donations.

The results of Study 2 replicate the pattern found in the previous studies, while measuring individual differences in the participant’s natural ongoing moods, as opposed to experimentally manipulated ones. Once again, the recipient’s identifiability significantly interacted with the participant’s mood in predicting donations, such that the Identifiable Victim Effect was more pronounced during negative moods. Moreover, the results of the present study provide initial evidence of the role played by emotions toward the victims in explaining the interaction between mood and identifiability on donations. When in a positive mood, it seems, people are less emotionally engaged with the specific case of the identified victim, and emotions toward such individuals are attenuated. This idea is discussed further in the following section.

## General discussion

In this paper, we set out to examine the role of positive and negative mood in donation decisions with regard to identified victims and to general needs. Based on previous research on the identifiable victim effect, we hypothesized that mood may interact with the identifiability of the helping target, such that the identified victim effect would be attenuated under a positive mood. The results of two studies show a significant interaction between mood and identifiability. Study 1 (as well as the pilot study) demonstrates causal relationships between mood and the identifiability effect, in that they replicated the same pattern, with different manipulations to induce moods, and eliciting real donations as well as a hypothetical willingness to donate. In both studies, donations to identified single victims exceeded donations to a general cause (as represented by a group of unidentified victims), in the Negative Mood manipulations and in Study 1 in the control condition as well—while participants in the Positive Mood conditions did not show this preference, and even tended to donate more to general causes, rather than to specific identified recipients (although not significantly so). The results of Study 1 are in line with previous research, in that they demonstrate that people in a negative mood act similarly to people in a control condition, while a positive mood yields a different behavioral pattern [e.g. in evaluating the affective qualities of future activities, 6; in paying selective attention to rewarding information, 29]. Finally, in Study 2, individual differences in people’s natural moods interacted with the recipient’s identifiability—demonstrating that the identified victim effect was attenuated when subjects were in a positive mood.

Our findings are consistent with the research on mood and generosity, which suggests that people who are in a positive mood tend to be more inclined to help than those who are in a negative or neutral mood—albeit only when this is not expected to spoil the helper’s mood [30, 47, 57]. Comparing participants’ moods before and after reading about victims in need (using a visual analog scale without digits), Kogut and Ritov [45] found that the participants’ mood worsened more after being presented with a single identified victim than after being presented with a group of victims experiencing the same need (whether they were identified or not). Thus, people who are in a positive mood may avoid getting involved in the story of the identified victim, to avoid the anticipated mood change. Conversely, being presented with a general need (such as a charitable organization) is less emotionally wrenching, and people may anticipate positive emotions, such as a warm glow, when donating to such good causes [58], and fewer negative ones (such as distress). The results of Study 2 lend support to this idea by demonstrating that participants who were in a better mood expressed weaker emotions toward identified victims, while emotions toward the victims in the General Need condition were not attenuated by mood. Conversely, participants who were in a worse mood exhibited greater emotional reactions toward identified victims than toward unidentified, generic victims. It should be noted, however, that the self-reported emotions toward the victim(s) were assessed *after* the donation decision, and may have served to justify the subject’s behavior, rather than causing it. Future research should directly examine this question, by measuring emotions before and after the decision, as well as by manipulating them.

According to research on prosocial behavior, in order to help others people must first recognize the other’s need and pay real attention to it. Being distracted by external factors such as noise, haste, or competing stimuli [59, 60] or internal factors (such as having to deal with one’s own concerns, losses and needs), constrains the amount of attention and energy one can spare for others, resulting in decreased helping [61]. For example, recent research on the effect of the experience of physical needs on people’s donation decisions demonstrates that experiencing a continuous need (such as hunger) decreases the inclination to help others in need—even when those needs echo those of the responder’s [62]. People in a negative mood are expected to pay less attention to the needs of others and to be focused more on their own concerns. The need of a single identified target is more salient, and therefore more likely to attract the attention of people who are in a negative mood more than a general target. In addition, being told about a specific identified target may induce perceptions of similarity between the perceiver and the victim, by providing a tangible target for one’s feelings [51]. Most importantly, the identified target provides a concrete opportunity to help, feel meaningful and to improve their negative mood. In this research, we have made a first attempt to examine emotions toward the victim(s) as an explanation for the interaction between mood and identifiability in donation decisions, since the identifiability effect is largely explained by the spontaneous emotional reaction toward individual identified victims. This explanation is in line with the *mood maintenance* notion, suggesting that people who are in a positive mood are reluctant to contemplate situations that are expected to ruin their mood. However, there may be other mechanisms underlying the donation pattern that we observed, such as selective attention and perceived efficacy. Future research is needed to directly examine such possible mechanisms.

There are other limitations of the present research. One is that the majority of participants in the samples were women, which may skewed the results, as there is evidence that women tend to be more altruistic than men [34, 63], and may be more affected by mood-enhancing manipulations than men [64]. Future research is needed to examine whether the pattern that we found will replicate in samples that are more balanced in terms of participants’ gender. Second, although we rely on mood maintenance theory to explain our results, we did not measure participants’ mood after the donation decision, to track the expected change in donors’ mood after the donation (specifically, the relief that participants in negative mood may have experienced). Future research is therefore needed to directly measure the actual change in affect in the wake of the donation decision, for participants in various moods.

Third, the present research is focused on positive and negative moods. In future studies it would be important to examine various other specific emotional states (such as anger, guilt, pride, etc.) and their possible interaction with the identifiability of the recipient. Lastly, in the present research we compared a single identified victim with an organization (general need), since these two presentations are the most common in real-life donation requests. However, our manipulation involved a comparison between a single identified victim and several unidentified victims. Future research is needed to directly compare the effect of mood on the identifiable victim effect in relation to a single identified victim versus a single unidentified one. Based on previous research [1, 2]—which consistently shows that a single identified victim evokes a stronger emotional reaction and elicits more donations than a single unidentified victim or a group of people (regardless of their identifiability)—we expect that mood will have a similar effect on donations in these new conditions (a single unidentified victim or a group of identified victims)—such that people in a positive mood will be more inclined to donate to help victims who tug less strongly on one’s emotional strings, compared with individual identified victims.

In light of the findings regarding people’s unconscious preference to help single identified victims, research in the past decade has struggled to find ways to increase donations to save large numbers of people, or to help multiple targets. Small, Loewenstein and Slovic [4] attempted to counteract the spontaneous reaction toward identifiable victims by telling participants about the effect, and encouraging them to think analytically about the greater value involved when more lives are at risk. They found that while engaging in a deliberative mode of thought decreases contributions to single victims, it does not increase contributions to entire groups of victims. That said, Hsee and colleagues [65] did find that asking donors to specify the amount needed to help one individual in a group or crowd of people in need, just before asking them to decide how much to donate to help the entire group, increased donations to the group as a whole. Similarly, increasing the unity of a group by making it look more like a single entity than a group of many individuals [66] can boost the role played by emotions in decisions about the group. For example, donations to help children in need increase when the children are of the same family, compared to when they have no shared affiliation [67]. In a recent study Kogut, Slovic & Västfjäll [68] found that increasing the victims’ collectivist values may enhance participants’ caring for groups without reducing their donations toward single individuals.

The present study may therefore offer some practical strategies to make donations requests more efficient, by matching the type of the request to the emotional context. Our results reveal that approaching people while they are in a happy mood (e.g. at a celebration, or at festive events) may enhance donations to groups and to crowds in need, while in a negative emotional context (e.g. a memorial ceremony) presenting the case of a single identified victim may be more effective.

## Supporting information

S1 File Happy to help_pilotStudy_data. (SAV)

S2 File Happy to help_Study1_data. (SAV)

S3 File Happy to help_Study2_data. (SAV)
